# Design Principles for Neurorobotics

**DOI:** 10.3389/fnbot.2022.882518

**Published:** 2022-05-25

**Authors:** Jeffrey L. Krichmar, Tiffany J. Hwu

**Affiliations:** ^1^Department of Cognitive Sciences, University of California, Irvine, Irvine, CA, United States; ^2^Department of Computer Science, University of California, Irvine, Irvine, CA, United States; ^3^Riot Games, Los Angeles, CA, United States

**Keywords:** adaptive behavior, embodiment, learning, memory, neuromodulation, value

## Abstract

In their book “How the Body Shapes the Way We Think: A New View of Intelligence,” Pfeifer and Bongard put forth an embodied approach to cognition. Because of this position, many of their robot examples demonstrated “intelligent” behavior despite limited neural processing. It is our belief that neurorobots should attempt to follow many of these principles. In this article, we discuss a number of principles to consider when designing neurorobots and experiments using robots to test brain theories. These principles are strongly inspired by Pfeifer and Bongard, but build on their design principles by grounding them in neuroscience and by adding principles based on neuroscience research. Our design principles fall into three categories. First, organisms must react quickly and appropriately to events. Second, organisms must have the ability to learn and remember over their lifetimes. Third, organisms must weigh options that are crucial for survival. We believe that by following these design principles a robot's behavior will be more naturalistic and more successful.

## 1. Introduction

Neurorobotics is a powerful tool for testing brain theories and increasing our understanding of neuroscience. The robot controller is modeled after some aspect of the nervous system. Unlike human or other animal studies, the neuroroboticist has access to every aspect of this artificial brain during the lifetime of the agent. Therefore, the neuroroboticist can analyze and perturb the nervous system in ways that a neuroscientist cannot with present recording technology. Not only can a neurorobot be tested under laboratory conditions that are similar to those of an animal experiment in order to provide direct comparisons, but it can also be tested in more natural conditions to see how these brain functions might respond to real-world situations.

The actions of a neurorobot are embedded in its environment. By choosing an appropriate morphology, simple mechanical designs can perform complex functions by taking advantage of environmental features, thus alleviating slow, power-hungry nervous systems from having to make these calculations. This is known as morphological computation (Pfeifer and Bongard, 2006). Neurorobot designs can be degenerate, that is, they can contain multiple systems capable of performing the same functions (Edelman and Gally, 2001). In this way the agent can still survive in the environment should one system fail. Similar to many mobile operating systems, neurorobot computation can follow the brain architecture by having multiple processes run in parallel in an event-driven manner, continuously responding to concurrent events. These ideas have roots in behavior-based robots (Brooks, 1991; Arkin, 1998) and the design of neuromorphic hardware (Merolla et al., 2014; Davies et al., 2018).

To adapt to a changing environment a neurorobot must be able to learn, store and recall information. Memory systems in neurorobotics are particularly applicable in spatial memory for navigation and contextual memory for learning representations of the environment (Milford et al., 2016; Gaussier et al., 2019; Hwu et al., 2020). Success within dynamic environments, such as the real-world, requires the processing of risk, reward, and uncertainty by some notion of value and the ability to adapt (Oudeyer and Kaplan, 2007; Krichmar, 2008; Merrick, 2017). Through such systems, neurorobots are able to predict future events and adapt to changes in the environment.

The world is full of trade-offs and changing needs that require us to make choices. Incorporating behavioral trade-offs into neurorobots such as reward vs. punishment, invigorated vs. withdrawn activity, expected vs. unexpected uncertainty for attention, exploration vs. exploitation of choices, foraging for food vs. defending one's territory, coping with stress vs. keeping calm, and social interaction vs. solitary restraint can lead to interesting behavior in neurorobotics (Canamero et al., 2006; Hiolle et al., 2012; Krichmar, 2013; Lones et al., 2018). Many of these trade-offs are regulated by the neuromodulators and hormone levels in the brain.

In this article, we present a set of principles to take into consideration when designing neurorobots. They fall into three categories: 1) Embodiment and reactions, 2) Adaptive behavior, learning and memory, and 3) Behavioral trade-offs. Following these design principles can make neurorobots more naturalistic and more interesting. Many of the ideas put forth in this article are based on material from our forthcoming book (Hwu and Krichmar, 2022).

## 2. Embodiment and Reactions - Responding to the Here and Now

In this first set of neurorobotic design principles, we focus on what (Pfeifer and Bongard, 2006) called the “here and now.” These design principles are grounded in neuroscience and are focused on processes that respond to events. Even without learning and memory, these processes lead to flexible, adaptive behavior.

### 2.1. Embodiment

Brains do not work in isolation; they are closely coupled with the body acting in its environment (Chiel and Beer, 1997). Biological organisms perform *morphological computation*; that is, certain processes are performed by the body that would otherwise be performed by the brain (Pfeifer and Bongard, 2006). Moment-to-moment action can be handled at the periphery by the body, sensors, actuators, and reflexes at the spinal cord level. This allows the central nervous system, which is slower and requires more processing than the body or peripheral nervous system, to predict, plan, and adapt by comparing its internal models with current information from the body (Shadmehr and Krakauer, 2008; Hickok et al., 2011).

In biology, the morphology and behavior must fit within the organism's ecological niche. Therefore, the layout of its sensors and actuators, their resolution and range are tuned to meet the specific organism's needs (Ziemke, 2003). As an example, consider a toddler flailing his or her arms. The child's arms more easily move toward the front of the torso than the back. By chance the toddler's hand touches an object causing a reflexive grasping motion. This leads to the fingers, where most of the tactile sensors are located, touching the object. The child will then move this hand in its easiest direction, which tends to be toward the face, where a range of sensors for vision, olfaction, and taste receptors reside. Comparing that with the embodiment and design of an insect or a fish, it is clear that these design implementations are specific to the organism's niche. Attention to these environmental details can provide guidance for the design of neurorobots. The form of the organism's body shapes its behavior and its brain function. This requires a brain and body that is engaged with the environment, which is what we should strive for in designing our neurorobots.

For many tasks that we carry out with ease, our brains are too slow to sense, process and move. For example, skiing down a hill or catching a wave on a surfboard happens too fast for our central nervous system to position the body appropriately. But the form and compliance of the body can position itself properly and adjust itself to perturbations without brain control. This is morphological computation in action.

**Figure 1:**
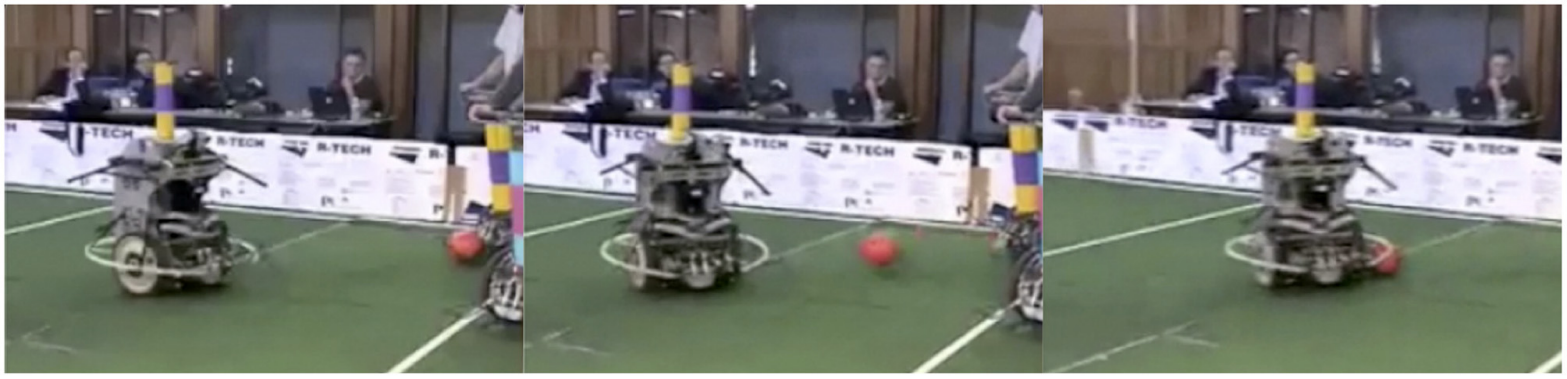
Video sequence showing robot capturing a soccer ball using morphological computation. A pliant plastic hoop around the Segway robot allowed trapped the soccer ball, allowing the slower IR sensors and camera to confirm that a soccer ball was caught.

Trapping a soccer ball is another example of morphological computation. In a RoboCup tournament, the Segway soccer team from The Neurosciences Institute solved this difficult sensorimotor problem with a very cheap design (Fleischer et al., 2006). On a large playing field it was nearly impossible for the robot to catch a fast-moving soccer ball, given that the Segway was large and cumbersome and had a slow camera frame rate and slow IR sensor refresh rate. Soccer balls would bounce off the robot before it had a chance to respond. After much trial and error, the team used plastic tubing that was fastened around the robot's body like a hula-hoop at just the right height (see Figure 1). Any ball that was passed to the Segway robot was trapped by the tubing, giving the robot time to use its camera and IR proximity sensors to place the ball in its kicking apparatus. In a sense, this is what humans do when playing soccer. They use soft pliant materials angled appropriately to soften the impact of a ball coming toward them. Many actions like these take place without much thought (i.e., brain processing).

By putting more emphasis on designs that exploit the environment, we can offload some of the control from the cognitive robot's central nervous system onto the body itself. This should allow the robot to be more responsive to the environment and more fluid in its actions. In addition, it frees up the nervous system to put more emphasis on planning, prediction, and error correction rather than reflexive movements. Too often cognitive neuroscientists forget that the body and the peripheral nervous system are performing many vital, moment-to-moment behaviors and tasks without central control. Even functions that are thought to be purely mental have a basis in embodiment. For example, it has been argued that interactions in which a person needs to understand another is an embodied process rather than an internal simulation (Gallagher, 2001).

### 2.2. Efficiency Through Cheap Design

Cheap design means finding the simplest solution to the challenge the robot is facing. One way to do this is by exploiting the environment. For example, winged insects and fast swimming fish exploit their environment by creating vortices with their wing beats or fin movements, which causes additional thrust and more energy to come out than the animal put in. Most of our touch receptors are where we need them most, at our fingertips. It would be wasteful to have this fine level of resolution on the back of our hands or arms. This is what is meant by cheap.

For example, in legged locomotion, roboticists have put much time and effort in creating robust controllers for legged locomotion. The importance of the cheap design principle can be observed when comparing the biped locomotion of passive dynamic walking robots to sophisticated humanoid robots, such as Honda's Asimo or Aldebaran's NAO. Passive dynamic walking robots exploit gravity, friction, and the forces generated by their swinging limbs (Collins et al., 2005). As a result, they require very little energy or control to move (see Table 1). In contrast, robots such as Asimo need complex control systems and long-lasting batteries to achieve the same result. Although these passive walkers are not necessarily biologically inspired, once the engineers or artists implement a design that minimizes energy expenditure, the gait looks very natural.

**Table 1:** Energy consumed during legged locomotion (Collins et al., 2005). Unit weight per unit distance.

**Agent**	**Energy consumption**
Asimo	3.23
Cornell biped	0.20
Humans	0.20

However, it should be stressed that passive dynamics and morphological computation are not enough to support a complete range of natural behaviors. Rather it frees up the system from expending energy and computational resources on some functions, while allowing it to concentrate on other functionality. Saving energy is a recurring theme in biology since biological organisms are under tight metabolic constraints (Beyeler et al., 2019; Krichmar et al., 2019). However, there is a trade-off that comes with efficiency. For example, it is more efficient to walk on four legs, but then arms are not available for manual dexterity and gestures, which is important for bipedal organisms. In a complete system, passive control is closely coupled with spinal cord reflexes, which in turn are in close communication with motor cortex and other areas of the brain. These issues should be taken into consideration when designing neurorobots.

It is not only the body that follows the principle of cheap design: brains do as well. Biological systems are under extreme metabolic constraints and need to represent information efficiently. Therefore, the nervous system must encode information as cheaply as possible. The brain operates on a mere 20 watts of power, approximately the same power required for a ceiling fan operating at low speed (Krichmar et al., 2019). Although being severely metabolically constrained is at one level a disadvantage, evolution has optimized brains in ways that lead to incredibly efficient representations of important environmental features that are distinctly different from those employed in current digital computers. The brain utilizes many means to reduce its functional metabolic energy use. Indeed, one can observe at every level of the nervous system strategies to maintain high performance and information transfer while minimizing energy expenditure.

At the neuronal coding level, the brain uses several strategies to reduce neural activity without sacrificing performance. Neural activity (i.e., the generation of an action potential, the return to resting state, and synaptic processing) is energetically very costly, and this drives the minimization of the number of spikes necessary to encode the neural representation of a new stimulus. Such sparse coding strategies appear to be ubiquitous throughout the brain (Olshausen and Field, 2004; Beyeler et al., 2019). Efficient coding reduces redundancies and rapidly adapts to changes in the environment. At a macroscopic scale, the brain saves energy by minimizing the wiring between neurons and brain regions (i.e., number of axons) and yet still communicates information at a high level of performance (Laughlin and Sejnowski, 2003). Information transfer between neurons and brain areas is preserved by a small-world network architecture, which reduces signal propagation (Sporns, 2010). These energy-saving ideas should be taken into consideration in constructing neural controllers for robots, which like biological organisms have limited energy resources. Moreover, many of these strategies could inspire new methodologies for constructing power-efficient artificial intelligence.

### 2.3. Sensory-Motor Integration

In the brain, the sensory and motor systems are tightly coupled. An organism or robot may get new sensory information that causes an action. Each action then creates new sensory information. Neurorobots can take advantage of this tightly coupled loop. For example, figure-ground segregation is a difficult computer vision problem in which a scene of static objects needs to be recognized from the background (e.g., a small toy block sitting on a similarly colored table). However, segmentation can be facilitated by sensorimotor integration in a very natural way as was demonstrated in a robot experiment by Fitzpatrick and Metta (2003). In their study, the robot's hand moved until it happened to hit the toy block, triggering motion detector responses in its visual system. In this way the robot's motor system generated sensory information, both visual and tactile, which led to the unexpected recognition of an object on the table. In the nervous system, a copy of the action, called a motor efference copy, is fed back to the brain. It creates an expectation that can be used to error check the movement and the expected sensory experience. Because hitting the toy creates a violation of both tactile and visual sensory expectations, the toy block is easily differentiated from the table.

It is important to emphasize how much the sensory and motor nervous systems are intertwined. Too often neuroscientists study these systems separately, but they are highly interconnected and work in concert. Although there are brain areas specialized for sensing such as auditory cortex and visual cortex, and there are areas of the brain devoted to action such as the motor cortex, most of the cortex is associational and cannot be called exclusively sensory or motor systems. These associational cortical areas are highly interconnected and the delineation between perception and action becomes blurred (Fuster, 2004). The parietal cortex receives multimodal sensory inputs and is important for planning movements. By multimodal we mean that the brain area receives more than one sense: auditory, olfactory, taste, visual, touch, or vestibular. The frontal cortex also receives multimodal inputs and is important for decisions, control of actions, and action selection. These multimodal association areas have direct influence on what we perceive and how we move.

### 2.4. Degeneracy

Degeneracy is the ability of elements that are structurally different to perform the same function or yield the same output (Edelman and Gally, 2001). To be fault tolerant and flexible a system's architecture should be designed such that different subsystems have different functional processes and there is an overlap of functionality between subsystems. In this design, if any subsystem fails the overall system can still function. This is different from redundancy, in which an identical system copy is kept in case there is a system failure (e.g., redundant array of independent disk [RAID] computer memory systems). Degeneracy appears throughout biology, from low-level processes such as the genetic code and protein folding to system-level processes such as behavioral repertoires and language. For example, there are four nucleotide bases in DNA (thymine, cytosine, adenine, and guanine). It takes three bases to encode an amino acid, which is the building block of proteins. This means that there are potentially 4^3^ or sixty-four possible combinations, but only twenty amino acids make up the proteins found in the human body. In many cases, different triplets encode the same amino acid. Therefore, the genetic code is considered degenerate. As a result, the genetic code is fault tolerant to mutations. The heterogeneity of neuron types within and between brain regions, as well as between organisms is another example of degeneracy. For instance, the nervous system has numerous cell types which can be distinguished by their anatomy, connectivity or firing behavior (Ascoli et al., 2007; Wheeler et al., 2015). Furthermore, organisms like the nematode C. Elegans have neurons that don't fire action potentials (Sarma et al., 2018). Despite this variability, these neuronal elements communicate with sensors, actuators, and other brain regions that have similar properties and often operate in the same environment. At the other end of the biological spectrum is communication. We have an almost infinite number of ways to communicate the same message. The same message could be communicated through voice, text, email, Morse code, gesture, or facial expressions. Degeneracy and variability is not only important to demonstrate for biological realism, it also leads to robustness and fault tolerance when operating in noisy, dynamic environments.

**Figure 2:**
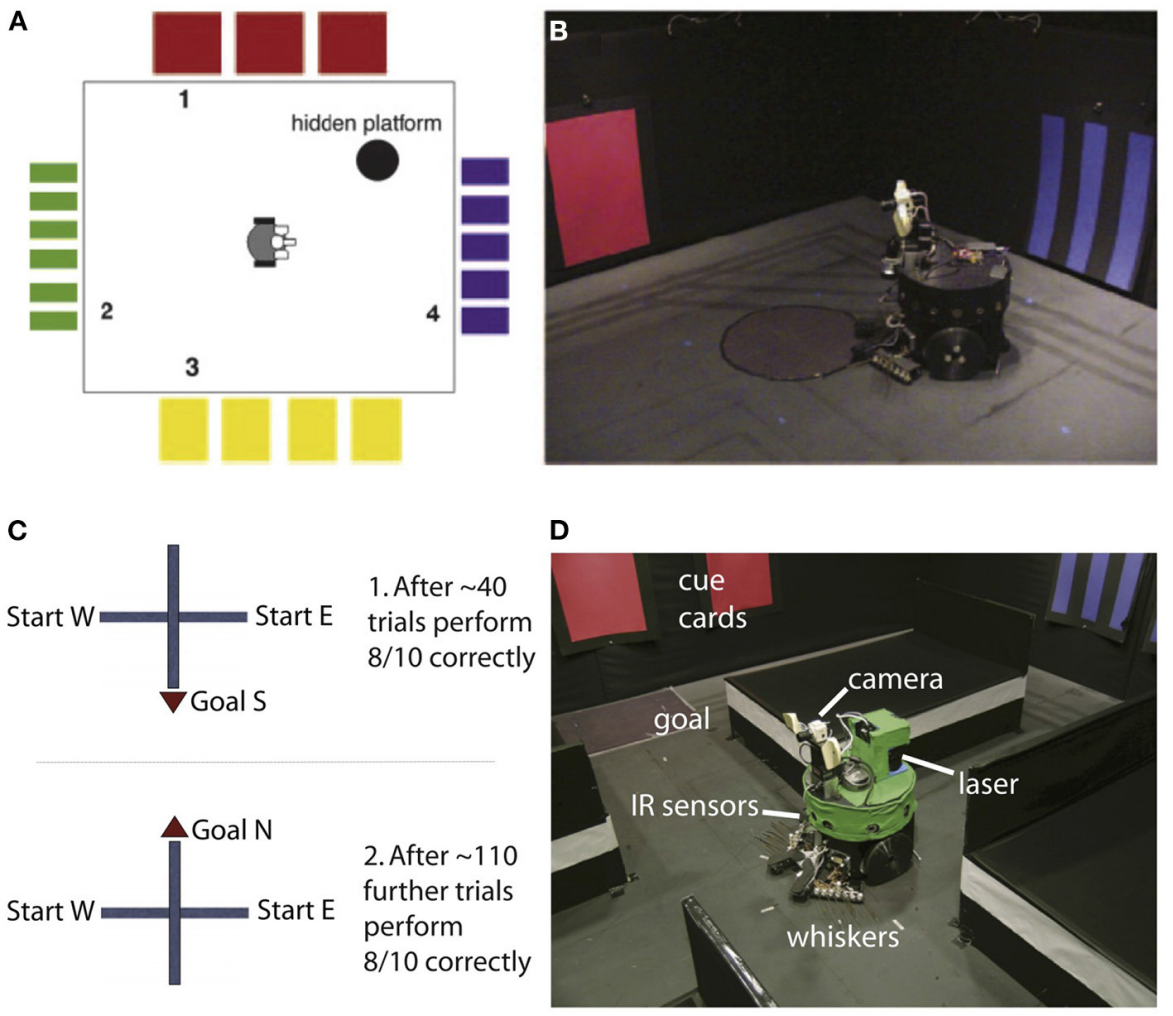
Darwin X experiments for spatial and episodic memory. **(A,B)** Experimental setup for a dry version of the Morris water maze. Adapted from Krichmar et al. (2005). **(C,D)** Experimental setup for for place learning with the plus maze. Adapted from Fleischer et al. (2007).

Degeneracy at multiple levels was nicely demonstrated by the neurorobot Darwin X, which was used to demonstrate spatial and episodic memory (Krichmar et al., 2005; Fleischer et al., 2007). Darwin X solved a dry version of the Morris water maze and a place-learning version of a plus maze (see Figure 2). In the Morris water maze, a rat swims through murky water until it finds a platform hidden beneath the surface. After several days of exploration, the rat will swim directly to the platform from any starting location. If the hippocampus is damaged, the rat cannot learn the location of the platform. In the dry version, a reflective piece of paper was the proxy for the platform. It was the same color as the floor, so the robot could not see the platform but it could “feel” when it was on the platform by means of a downward-pointing light sensor.

Darwin X had an extensive model of the hippocampus formation and its surrounding cortical regions. Similar to a rodent, as the robot explored its environment, hippocampal place cells emerged. In the Darwin X experiments, combinations of place cells were used to plan routes to goals. The robot's behavior and neural activity were directly compared with rodent experiments. Like the rat, place cells could be used to predict not only the current location of the robot but also the location from which the robot came and the location to which it was heading. In the robot experiments, several levels of degeneracy emerged.

#### Degeneracy at the Neuronal Level

Because the neuroroboticists were able to track every neuron in Darwin's simulated nervous system, they were able to trace the neuronal activity that led to hippocampal place activity. Although hippocampal place activity was similar on different trials when the robot passed through the same location on the same heading, the neuronal activity leading to that neuron's place activity on a given trial differed dramatically. That is, different neural activation patterns led to the same hippocampal place cell outcome.

#### Degeneracy at the Systems Level

Darwin X received sensory input from its camera (vision), whiskers (somatosensory), compass (head direction), and laser range finder (depth/distance). Darwin X's spatial memory was multimodal and degenerate. Even when one or more of its sensory modalities were lesioned, Darwin X's behavior and place cell activity remained stable. Different sensory pathways led to the same outcome of knowing where Darwin was located.

#### Degeneracy at the Behavioral Level

In the Morris maze task, nine different Darwin X subjects, which consisted of the identical robots with slightly different nervous systems due to variations in initial synaptic connection probabilities, solved the same spatial navigation task in unique ways. Some subjects bounced off the “red” wall to the hidden platform, some bounced off the “blue” wall, and others went directly toward the platform location. The proficiency of each subject differed as well. Some were better learners than others. However, despite their idiosyncrasies they all shared the same outcome of solving this task.

### 2.5. Multitasking and Event-Driven Processing

Cognitive scientists tend to study the brain in a serialized fashion by focusing on one subsystem at a time, be it a type of memory or a specific perceptual effect. But intelligence emerges from many processes operating in parallel and driven by events. We (humans and other organisms) are multitaskers, and to multitask we must do things in parallel. The brain is the ultimate event-driven, parallel computer. There is no overarching clock as in computer architectures. Rather the brain responds to events when they happen. Therefore, neurorobots should have a multitask design that responds to multiple, asynchronous events in a timely manner.

Neurons throughout the brain are responding simultaneously to multiple events. Although the different parts of the brain may be acting somewhat independently, they are highly interactive. The sensory system is telling the motor system what it senses, and the motor system is telling the sensory system what its last action was. This brain analogy can be extended to the whole organism, in which control is parallel, asynchronous, and spatiotemporally matched with the real world.

A classic way of studying cognitive science was a serial process of “sense, think, and act,” which guided many artificial intelligence robot designs. In response to this, Rodney Brooks and Ron Arkin developed behavior-based robots that responded asynchronously to events (Brooks, 1991; Arkin, 1998). There are parallels between the subsumption architecture's layered hierarchical design and the nervous system. Neuroscientist Larry Swanson proposed a basic plan for the nervous system that somewhat follows this design; a four-component functional systems model with a motor system controlling behavior and visceral functions (i.e., internal organs and bodily functions), whose output is a function of activity in sensory, cognitive, and behavioral state systems, with feedback throughout (Swanson, 2007). It should be noted that in this view the central nervous system is only one component. Areas that regulate basic behaviors and internal monitoring are subcortical.

**Figure 3:**
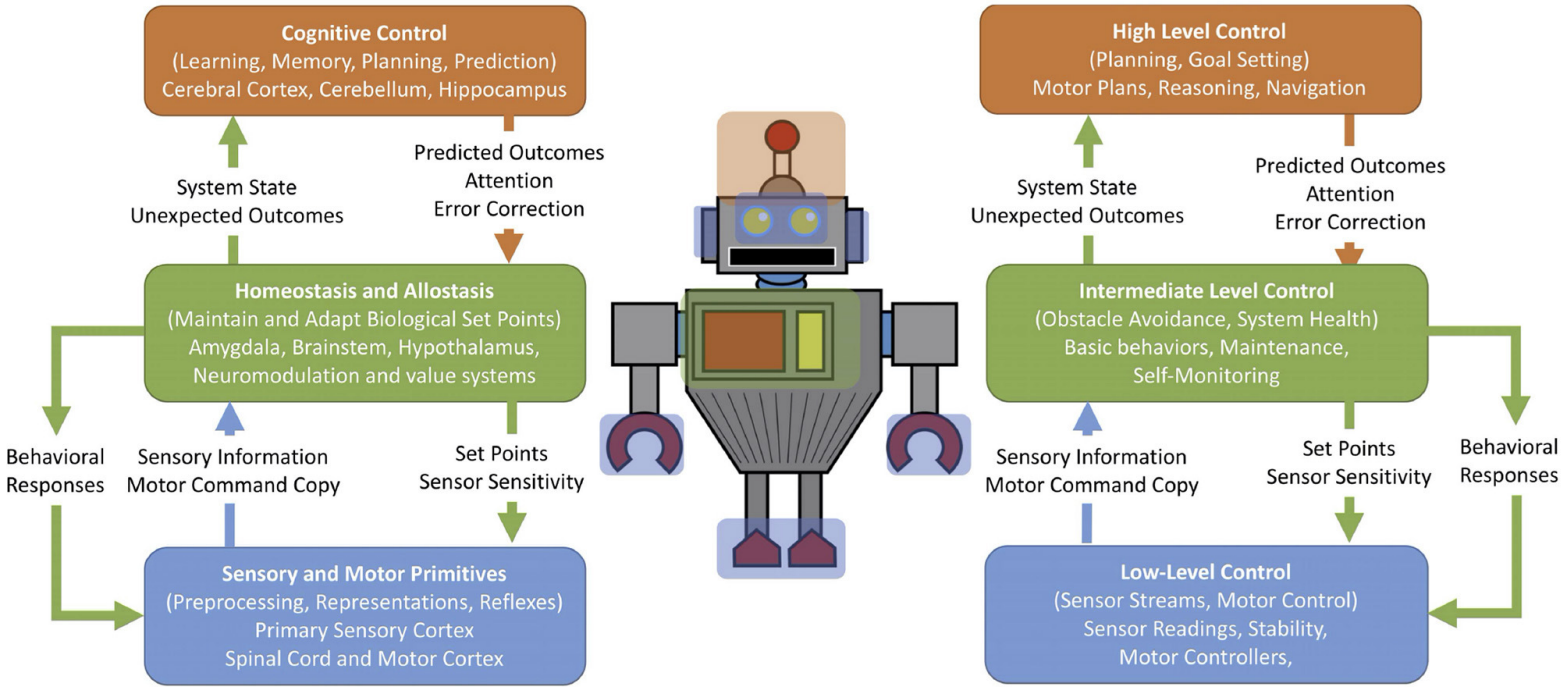
Schematic for self-monitoring systems in biology and in engineering. On the left are terms and regions derived from neuroscience. On the right are terms adapted from autonomous robots but could be applied to many embedded systems. Blue: low-level sensory processing and motor control. Green: homeostasis, maintenance, and monitoring. Orange: high-level planning, adapting, and goal-driven behavior. Adapted from Chiba and Krichmar (2020).

Correlates of the subsumption architecture can be observed in the brain's structure. Prescott et al. (1999) suggested that the hierarchical, layered subsumption architecture could describe the neural mechanisms of defense for the rat. For example, the lower levels were reactive and included withdrawal, startling, and freezing. The higher levels subsumed the lower levels by suppressing behavior or predicting outcomes through conditioning. Sensory input provides stimuli that can trigger behavioral responses. Another example involves self-monitoring systems in the nervous system (Chiba and Krichmar, 2020). Figure 3 shows an overview of this architecture with brain systems on the left and the corresponding robot control systems on the right. There is low-level control for sensor processing and motor reflexes. The autonomic nervous system may subsume these lower levels to maintain homeostasis or adapt set points. Higher levels can set states or context that may shape responses.

Multitasking and event-driven processing is prevalent in current technology due in part to the ubiquity of real-time and embedded systems. Most modern computing devices, including smartphones, desktop computers, onboard automotive computers and entertainment systems, have parallel processes to handle asynchronous events. Neuromorphic computing architectures developed by researchers and major chip companies such as IBM and Intel are asynchronous and highly parallel, and they are composed of many computing units that act like neurons (Merolla et al., 2014; Davies et al., 2018). This architectural design also follows the *Efficiency through Cheap Design* principle described in Section 2.2. Neuromorphic hardware architectures use orders of magnitude less power than conventional computing by not relying on a synchronous clock and using spiking elements (Krichmar et al., 2019). A neuron uses most of its energy when it fires an action potential and when an action potential is processed at the synapse. Because neurons do not fire often (in the typical range of 10–100 Hz), the nervous system is in low-power mode between spikes. This idea was not lost on most neuromorphic hardware designers. Furthermore, communication bandwidth is reduced because information is sent only when there is a spike event.

## 3. Adaptive Behavior - Learning and Memory

Adaptation requires learning and remembering what was learned so that it can be applied in the future. Motivation is a key driver of learning. Motivators take many forms, which are called value systems. Another key aspect of adaptive behavior is the ability to predict future events. This requires building up a memory of expectations and the ability to adjust when expectations do not meet the current situation.

### 3.1. Learning and Memory

Unlike artificial systems, our brains allow us to learn quickly, incrementally, and continuously. With just a few presentations of something new, we can learn to recognize an object or situation or even learn a new skill. When we learn something new, we do not forget what we have learned previously. Moreover, we can take what we learn from one situation and apply it to another. On the other hand, artificial learning systems struggle under these situations, suffer from catastrophic forgetting of previous learning when something new is learned, and have difficulties generalizing learning from one task to another.

A brain region important for learning and memory is the hippocampus. The hippocampus is necessary to learn new memories and to consolidate those new experiences into long-term memories that can last a lifetime. The hippocampus can rapidly learn new autobiographical and semantic information, sometimes in the first experience (i.e., one-shot learning). Over time, this information becomes consolidated in the rest of the brain. Having a rapid learning system that can interact with a slower long-term storage area, which has been called complementary learning systems (McClelland et al., 1995; Kumaran et al., 2016), is thought to be the means by which our brains overcome catastrophic forgetting (i.e., forgetting previously learned information when learning new information). This aligns with another memory model, known as the hippocampal indexing theory (Teyler and DiScenna, 1986), which states that memories in the form of neocortical activation patterns are stored as indices in the hippocampus that are later used to aid recall. Although this may be an oversimplification, the notion that the hippocampus and medial temporal lobe integrates multimodal information from the neocortex makes sense and is backed by experimental evidence.

Our memories have context, and this contextual information can help us generalize when we encounter novel yet similar situations. In the literature this is called a schema, which is the memory of a set of items or actions bound together by a common context (van Kesteren et al., 2012). For example, if you are in a restaurant, you expect to see tables, chairs, a menu, waiters, and so forth. If you go to a new restaurant, that common context information can be used to rapidly consolidate the novel information into the restaurant schema. This requires mental representations that are flexible enough to learn tasks in new contexts and yet stable enough to retrieve and maintain tasks in old contexts (Carpenter and Grossberg, 1987). Tse et al. (2007) demonstrated this by training rats on different schemas, which were collections of associations between different foods and their locations in an enclosure. They found that the rats were able to learn new information quickly if it fit within a familiar schema. Additionally, the rats were able to learn new schemas without forgetting previous ones. The hippocampus was necessary for learning schemas and any new information matching a schema. A subsequent study showed increased plasticity in the medial prefrontal cortex (mPFC) when information was consistent with a familiar schema (Tse et al., 2011). However, the hippocampus was not necessary to recall these memories, even after a short time period (e.g., 48 h). This challenged the idea of complementary learning systems because new information could rapidly be consolidated in cortical memory under these conditions.

The Tse et al. (2007) schema experiment was replicated with a neural network model based on interactions between the hippocampus and the mPFC (Hwu and Krichmar, 2020), and later tested on a robot required to create and utilize a schema (Hwu et al., 2020). A contextual pattern of objects and locations projected to the mPFC, in which each individual neuron encoded a different schema. The ventral hippocampus (vHPC) and dorsal hippocampus (dHPC) created triplet indices of schema, object, and location. The vHPC and dHPC drove activity that eventually activated a place cell through a winner-take-all process in the action network. This action neuron caused the robot to go to that location in search of the cued object. Contrastive Hebbian learning (CHL) was used to make the association between schema, object, and location. CHL utilizes oscillatory epochs, in which the duration depends on the familiarity and novelty of information, to learn associations. This assimilation of new information has similarities to adaptive resonance theory or ART (Grossberg, 2013). The model contained neuromodulators to encode novelty and familiarity. For example, if an object is novel and the context is unfamiliar, a new schema must be learned. However, if an object is novel and the context is familiar, the object can be added to an existing schema.

The schema model was embedded on the Human Support Robot (HSR) from Toyota (Yamamoto et al., 2018) and given the task of finding and retrieving objects in a classroom and a break room. In a trial, the robot was prompted to retrieve an object, which required prior knowledge of the schema to which the object belonged and the location of the object. This caused the robot to navigate toward a location, recognize the object, grasp the object, and then return the object to its starting location.

**Figure 4:**
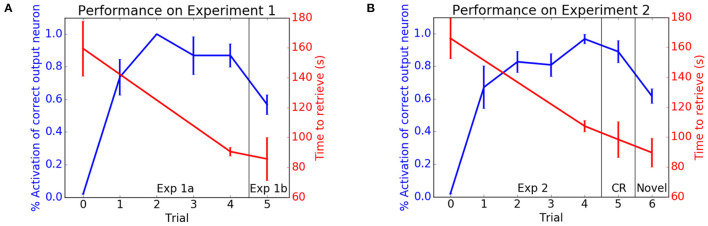
Performance on neurobotic schema experiments. The blue lines show the activation of the correct location neuron for a cued object. The red lines show the retrieval times. **(A)** Experiment 1. Performance in the classroom schema (Exp 1a) and retrieval of novel object (Exp 1b). **(B)** Experiment 2. Performance in the breakroom schema. CR denotes performance when returned to classroom. Novel denotes retrieval of a novel object. Adapted from Hwu et al. (2020).

In the first experiment, the HSR was placed in a room with typical classroom items (e.g., apple, bottle, computer mouse, book). Figure 4A shows the performance of the HSR retrieving classroom items. With each trial the number of correct places recalled increased and the time to retrieve an item decreased. After training and testing in the classroom, an original item was replaced by a novel item (Exp 1b in Figure 4A). Although the object was novel, the HSR knew it belonged in the classroom and was able to quickly consolidate this new information into the existing classroom schema.

In the second experiment, the HSR was placed in a room with typical break room items (e.g., apple, cup, banana, microwave oven). After training and testing, the classroom schema was tested again to see if the robot was able to maintain performance of prior tasks. As with the classroom, Figure 4B shows that the robot was able to learn this new break room schema without forgetting objects' locations in the classroom (CR in Figure 4B).

**Figure 5:**
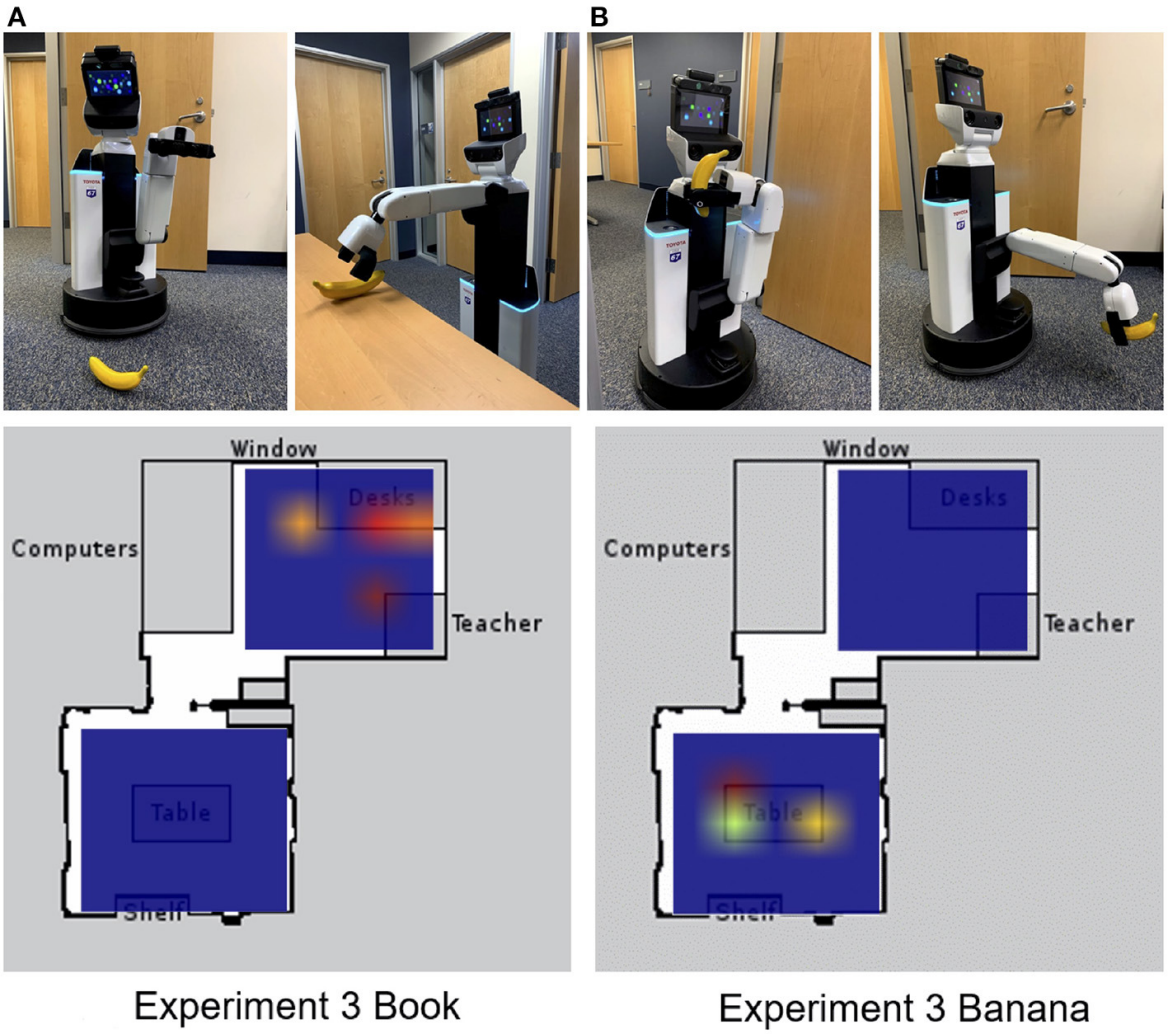
Cuing the robot on objects it had not retrieved before. Top: The robot was shown a banana. The robot then went to the break room to pick up the banana and navigated to the drop-off location to deposit it. Bottom: Heat map of action layer during experiment 3. **(A)** Cued to retrieve a book. **(B)** Cued to retrieve a banana. Adapted from Hwu et al. (2020).

In the third experiment, the HSR was tested to see whether schemas could help with the retrieval of items that it was never explicitly trained to retrieve. If the HSR was cued with a book, it searched for the book on the desk in the classroom because books are likely to be found in a classroom schema (see Figure 5A). If the HSR was cued by showing it a banana, the HSR searched for the banana in the break room first rather than in the classroom (see Figure 5B).

The neurorobotic schema experiments showed how context is tied to spatial representations via hippocampal interaction with the mPFC. Moreover, it demonstrates how ideas from memory models in the brain may improve robotic applications and issues in artificial intelligence, such as catastrophic forgetting and lifelong learning. A robot that has contextual memory could have applications for assistive technologies.

### 3.2. Value Systems

Robots should be equipped with a value system that constitutes a basic assumption of what is good and bad for its health and well-being. A value system facilitates the capacity of a biological brain to increase the likelihood of neural responses to an external phenomenon (Merrick, 2017). The combined effects of perception, experience, reasoning, and decision making contribute to the development of values in animals. Value can also be thought of as a measure of the effort one is willing to expend to obtain reward or to avoid punishment.

In addition to rewards and punishment, intrinsic motivation can be considered as a value system (Oudeyer and Kaplan, 2007). This can take the form of seeking novelty, fun, play or curiosity, that is, obtaining value for its own sake rather than to satisfy some need. For example, Oudeyer and colleagues created a robotic playground where a Sony AIBO dog with a motivation to explore and learn new things learned to manipulate objects (Oudeyer et al., 2007). The robot first spent time in situations that were easy to learn and then shifted its attention progressively to more difficult situations, avoiding situations in which nothing could be learned.

Neuromodulators are thought to act as the brain's value systems (Krichmar, 2008). Neuromodulators are chemicals that signal important environmental or internal events. They cause organisms to adapt their behavior through long-lasting signals to broad areas of the nervous system. Neuromodulators in the brain influence synaptic change (i.e., learning and memory) to satisfy global needs according to value.

To shape behavior, cognitive robots should have an innate value system to tell the robot that something was of value and trigger the appropriate reflexive behavior. From this experience the agent can learn which stimuli were predictive of that value and try to maximize the acquisition of good value while minimizing the acquisition of bad value. Many of these value-based robots employ models of the dopaminergic neuromodulatory system to shape behavior (Sporns and Alexander, 2002; Cox and Krichmar, 2009; Fiore et al., 2014; Chou et al., 2015).

Besides the dopaminergic reward system, there are multiple neuromodulators signal different value types (Doya, 2002; Krichmar, 2008). The serotonergic system is involved in harm aversion or impulsiveness (Miyazaki et al., 2018). The noradrenergic system signals oddball or unexpected events (Yu and Dayan, 2005). The cholinergic system is thought to increase attention to important features and at the same time to decrease the allocation of attention to distractors (Yu and Dayan, 2005). Acetylcholine and noradrenaline could be thought to signal intrinsic value by allocating attention and triggering learning (Avery and Krichmar, 2017). These systems interact with each other through direct and indirect pathways, and they all respond strongly to novelty by sending broad signals to large areas of the brain to cause a change in network dynamics resulting in decisive action.

**Figure 6:**
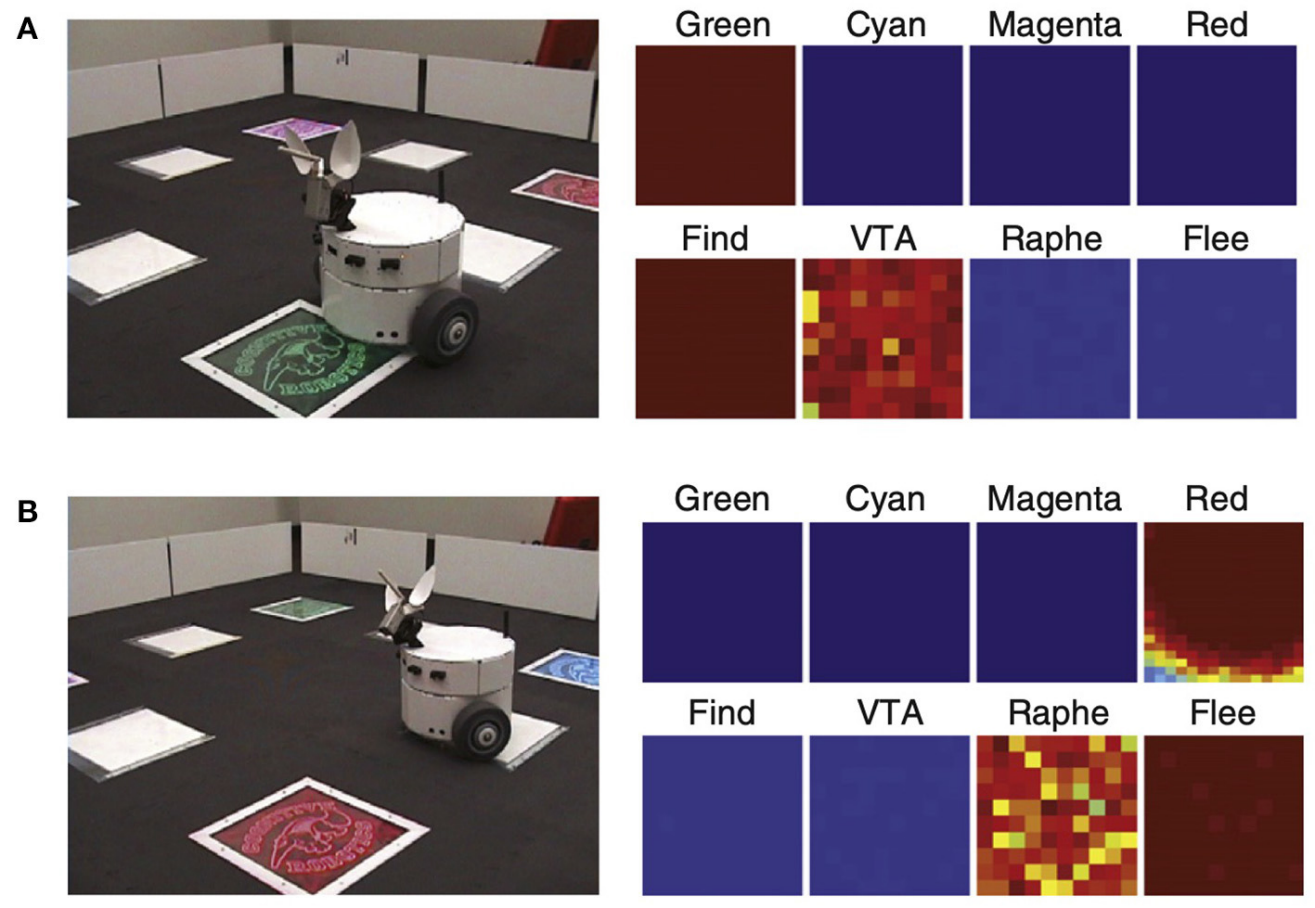
CARL robot in colored panel task. The panels could flash any of six different colors. One color, green, signaled positive value. Another color, red, signaled negative value. The remaining colors were neutral. Positive and negative signals were transmitted from the panel to a receiver on the bottom of CARL. **(A)** CARL during an approach or find response. The panels on the right show strong neuronal activity in its simulated green visual area, the dopaminergic system (VTA), and the find motor neurons. **(B)** CARL during a withdrawal or flee response. The panels on the right show strong neuronal activity in its simulated red visual area, the serotonergic system (raphe), and the flee motor neurons. Adapted from Cox and Krichmar (2009).

Introducing saliency into the environment can lead to attentional signaling. For example, the robot CARL was designed to test a computational framework for applying neuromodulatory systems to the control of autonomous robots (Cox and Krichmar, 2009). The framework was based on the following premises (Krichmar, 2008): (1) the common effect of the neuromodulatory systems is to drive an organism to be decisive when environmental conditions call for such actions and allow the organism to be more exploratory when there are no pressing events; and (2) the main difference between neuromodulatory systems is the environmental stimuli that activate them. In the experiment, two out of four objects were salient, and CARL learned the appropriate action for each (see Figure 6). Unexpectedly, a strong attentional bias toward salient objects, along with ignoring the irrelevant objects, emerged through its experience in the real world. The selective attention could be observed both in CARL's behavior and in CARL's simulated brain. These neurorobotic experiments showed how phasic neuromodulation could rapidly focus attention on important objects in the environment by increasing the signal-to-noise ratio (SNR) of neuronal responses. The model further suggested that phasic neuromodulation amplifies sensory input and increases competition in the neural network by gating inhibition.

**Figure 7:**
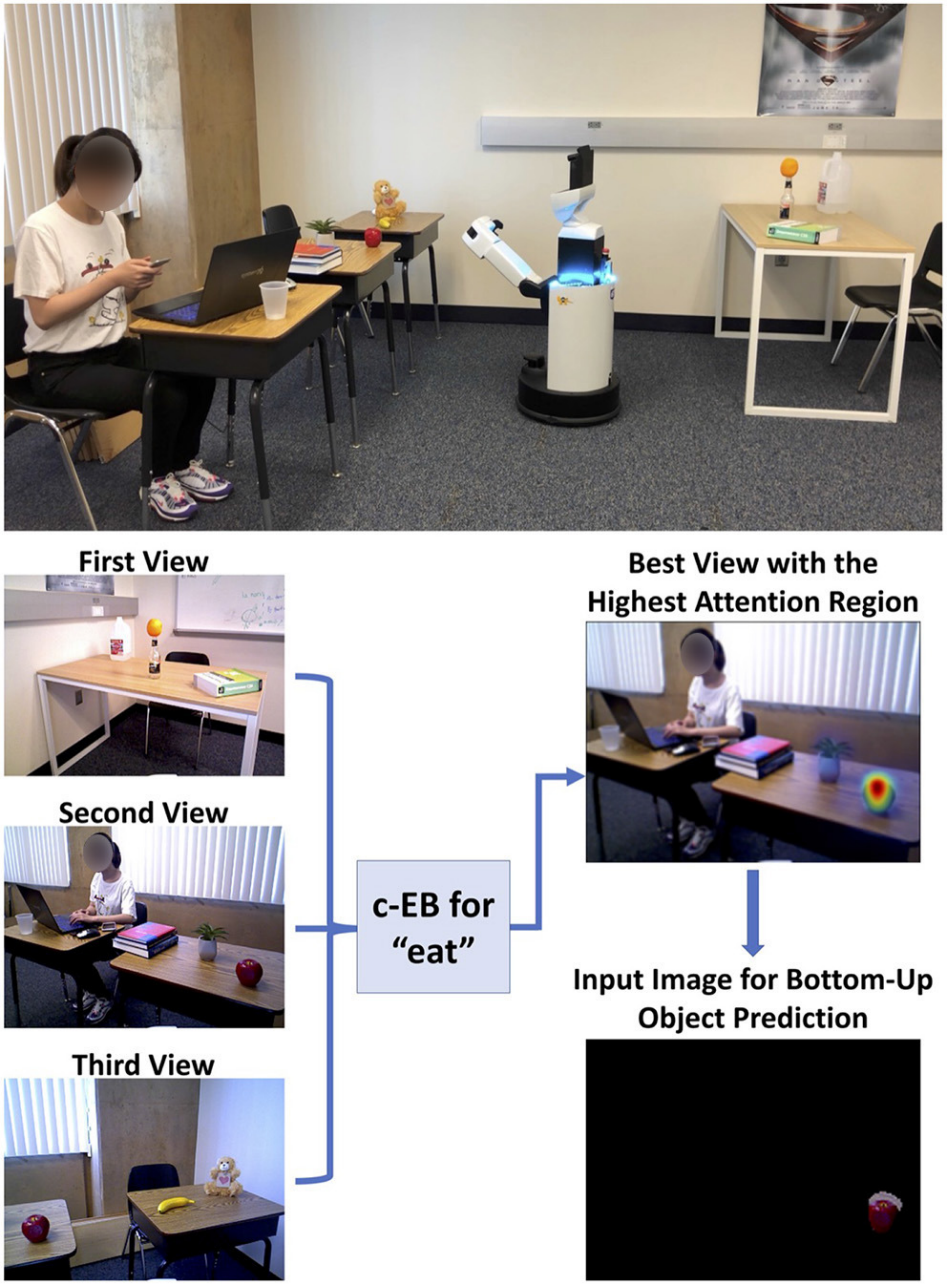
Toyota Human Support Robot implementation for the goal-driven perception model, including the top-down attentional search process for a guessed action “eat” based on three different real indoor views, to select the highest attention region for bottom-up object prediction. Adapted from Zou et al. (2020).

The neuromodulatory system also regulates attention allocation and response to unexpected events. Using the Toyota Human Support Robot (Yamamoto et al., 2018), the influence of the cholinergic (ACh) system and noradrenergic (NE) systems on goal-directed perception was studied in an action-based attention task (Zou et al., 2020). In this experiment, a robot was required to attend to goal-related objects (the ACh system) and adjust to the change of goals in an uncertain domain (the NE system). Four different actions (i.e., eat, work-on-computer, read, and say-hi) were possible in the experiment and each of them was associated with different images of objects. For example, the goal action “eat" might result in attention to objects such as apple or banana, whereas the action “say-hi" should increase attention to a person. During the experiment, the goal action changed periodically and the robot needed to select the action and object that it thought the user wanted on the basis of prior experience. The ACh system tracked the expected uncertainty about which goal was valid, and the NE system signaled unexpected uncertainty when goals suddenly changed (Yu and Dayan, 2005). High ACh activity levels allocated attention to different goals. Phasic NE responses caused a rapid shift in attention and a resetting of prior goal beliefs. The model demonstrated how neuromodulatory systems can facilitate rapid adaptation to change in uncertain environments. The goal-directed perception was realized through the allocation of the robot's attention to the desired action/object pair. Figure 7 shows the robot deciding which object to bring to the user. The bottom of Figure 7 shows views from the robot's camera as it correctly guesses that the user's goal is to eat. Its top-down attention system finds an appropriate object, which is an apple in this case.

One problem that remains unsolved in neurorobotics is that these artificial value systems are dissociated from the agent's body. Real pain, hunger, thirst, and fatigue drive a true value system. Without this connection to body-dependent drives, an artificial value system does not signal the immediacy of the agent's need and lacks to some degree the ability to shape the agent's behavior. It would be interesting to tie something like the robot's battery level to its hunger value. With faster-charging batteries or better solar cells this might be possible. An interesting step in this direction is the work on self-monitoring systems that can recognize drops in their performance, adapt their behavior, and recover. For example, Cully et al. (2015) developed a novel method for adapting gaits on a hexapod robot. In a sense, the robot controller had a memory of potential gaits. If one or more of the robot's legs were damaged, the robot would detect the damage, “imagine” different ways of moving, and then choose the new gait that it thought would work best under the new circumstances. In this way, the robot knew something was wrong and was able to adapt its behavior quickly without intervention.

### 3.3. Prediction

Predicting outcomes and planning for the future is a hallmark of cognitive behavior. Much of the cortex is devoted to predicting what we will sense or the outcome of a movement or what series of actions will lead to big payoffs. Thus, a neurorobot should strive to have these predictive capabilities. Through prediction and active inference, agents anticipate future sensory input based on prior experience. This minimizes free energy by predicting future outcomes so that they minimize the expenditures required to deal with unanticipated events (Friston, 2010). The idea of minimizing free energy has close ties to many existing brain theories, such as Bayesian brain, Predictive Coding, cell assemblies, Infomax, and the theory of neuronal group selection (Edelman, 1993; Rao and Ballard, 1999; Friston, 2010). In the theory of neuronal group selection (Edelman, 1993), plasticity is modulated by value. Value systems control which neuronal groups are selected and which actions lead to evolutionary fitness; that is, they predict outcomes that lead to positive value and avoid negative value. In this sense predicting value is inversely proportional to surprise.

Prediction is crucial for fitness in a complex world and a fundamental computation in cortical systems (George and Hawkins, 2009; Clark, 2013; Richert et al., 2016). It requires the construction and maintenance of an internal model. In a similar fashion, model-based reinforcement learning builds an internal model made up of the likelihood and expected value for transitions between states (Solway and Botvinick, 2012).

Prediction has been useful in developing robot controllers. For example, in a humanoid robot experiment it was shown that having a predictive model helped the robot make appropriate reactive and proactive arm gestures (Murata et al., 2014). In the proactive mode the robot's actions were generated on the basis of top-down intentions to achieve intended goals. In the reactive mode the robot's actions were generated by bottom-up sensory inputs in unpredictable situations. In another robot experiment the combination of model-based and model-free reinforcement learning was used in a sorting task (Renaudo et al., 2015). The robot had to push cubes on a conveyor belt. The model-based system improved performance by maintaining a plan from one decision to the next. However, the experiments suggested that the model-free system scales better under certain conditions and may be better in the face of uncertainty.

Jun Tani's group has developed several predictive robot controllers using recurrent neural networks (Tani, 2016; Ahmadi and Tani, 2019; Chame et al., 2020). For example, they trained a hierarchy of continuous time recurrent neural networks (CTRNN) to learn different movements. Learning was achieved via backpropagation through time (BPTT), a means to apply an error signal to a sequence of neural activities. A teacher guided a humanoid Sony QRIO robot through different behavioral tasks. The CTRNNs received visual information and proprioceptive joint angles from the humanoid robot. Important to the learning were the different timescales of the CTRNNs. Slower higher-level CTRNNs sent predictions to the faster lower-level CTRNN units. Prediction errors from the lower levels were propagated to the higher levels for adjustments. Movements that appeared repeatedly were segmented into behavioral primitives. These primitives were represented in fast context dynamics in a form that was generalized across object locations. On the other hand, the slow context units appeared to be more abstract in nature, representing sequences of primitives in a way that was independent of the object location. Tani (2016) speculated that this prediction multiple timescale hierarchy had similarities to the cortex. Fast responding motor primitives can be found in the primary motor cortex, and the slower prefrontal cortex sends top-down predictions to the primary motor cortex. Similarly, the primary visual cortex sends sensory information and prediction errors to the slower parietal cortex, which sends top-down predictions for the primary visual cortex. In this group's recent work, they show the potential for prototyping robotics agents, modeled after active inference from the free energy principle theory (Friston, 2010), for human-robot interaction and socially assistive robotics (Chame et al., 2020).

## 4. Behavioral Trade-Offs - Contextual Decision-Making

Biological organisms need to consider many trade-offs to survive. These trade-offs regulate basic needs such as whether to forage for food, which might expose oneself to predators, or hide in one's home, which is safer but does not provide sustenance. These trade-offs can be cognitive as in introverted or extroverted behavior. Interestingly, many of these trade-offs are regulated by chemicals in our brain and body, such as neuromodulators or hormones. These modulatory areas monitor and regulate environmental events. They send broad signals to the brain that can dramatically change behaviors, moods, decisions, etc. The brain can control these modulatory and hormonal systems by setting a context or making an adjustment when there are prediction errors (Chiba and Krichmar, 2020).

We discuss the neuroscience behind the trade-offs and neurorobots that incorporate these trade-offs. We consider these behavioral trade-offs to be neurorobotic design principles. By applying them to neurorobots, we may realize behavior that is more interesting and more realistic.

### 4.1. Reward vs. Punishment

Dopaminergic neurons have phasic responses that match quite well with a reward prediction error signal used to shape behavior (Schultz et al., 1997). What about punishment? One model suggested that tonic serotonin tracked the average punishment rate and that tonic dopamine tracked the average reward rate (Daw et al., 2002). They speculated that a phasic serotonin signal might report an ongoing prediction error for future punishment. It has been suggested that the serotonergic and dopaminergic systems activate in opposition for goal-directed actions (Boureau and Dayan, 2011). This trade-off between reward and punishment can be quite nuanced when invigoration of activity can lead to rewards and punishment can lead to inaction.

### 4.2. Invigorated vs. Withdrawn

The dopamine and serotonin systems also regulate a trade-off between invigorated novelty seeking and withdrawn risk-averse behavior. It has been suggested that serotonin modulates the desire to withdraw from risk, which can take place in social interactions or in foraging for food (Tops et al., 2009). One can imagine that too much withdrawal from society could lead to symptoms of depression.

Consider the open-field test that is used to measure anxiety in rodents (Fonio et al., 2009). Usually, when a mouse is placed in an unfamiliar open area it will first stay near the borders of the environment in which it might be concealed. The mouse may hide in a nest area if available. After some time the mouse decides the environment is safe and becomes curious. The mouse will then proceed to explore the environment by moving more and investigating the middle of the area. Serotonin levels can alter this behavior. For example, Heisler et al. (1998) showed that mice with increased serotonin spent less time in the center of the open-field arena. In contrast, cocaine, which increases the level of dopamine, increased locomotive activity and the exploration of novel objects (Carey et al., 2008).

**Figure 8:**
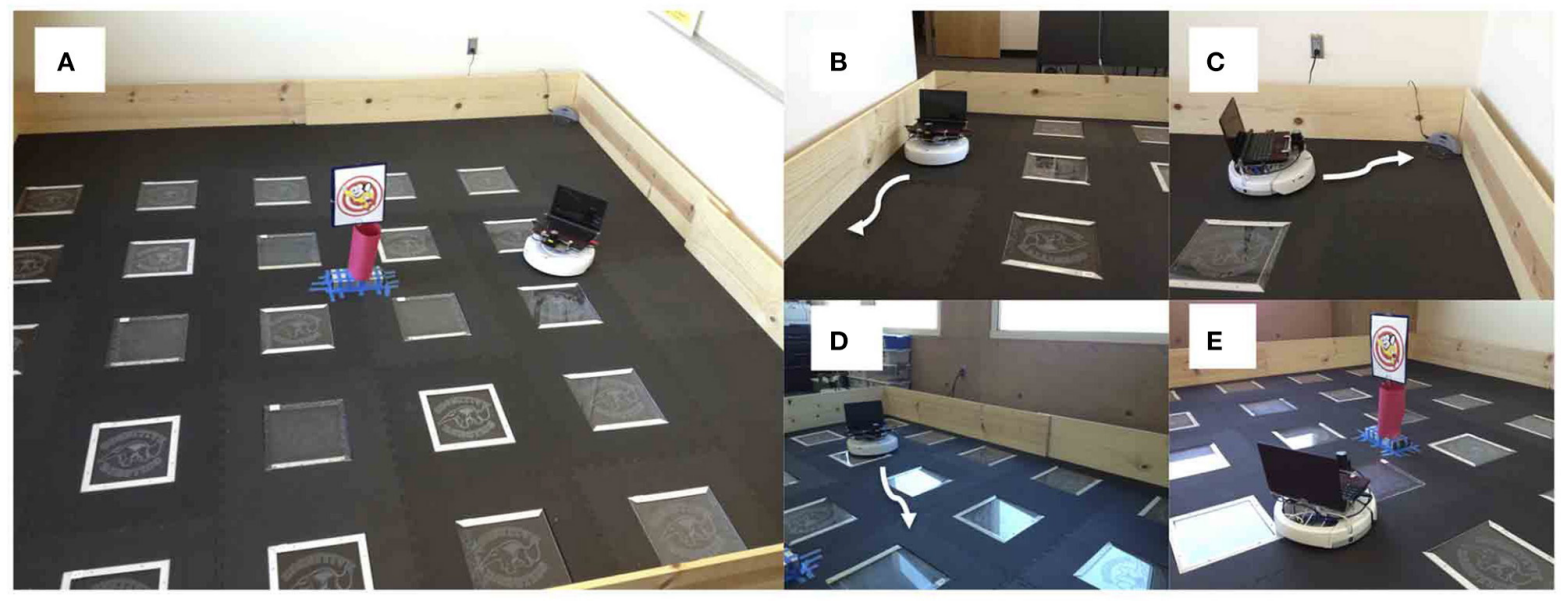
Neurorobotic experimental setup for invigorated and withdrawn behavior. Experiments were run on an iRobot Create. **(A)** Robot arena. The picture in the middle was a novel object for the robot to explore. **(B)** Wall follow behavior. **(C)** Find home behavior. Finding the robot's docking station. **(D)** Open field behavior. **(E)** Explore object behavior. Adapted from Krichmar (2013).

A neurorobot experiment took this trade-off into consideration by modeling the interactions between the serotonergic and dopaminergic systems (Krichmar, 2013). Figure 8 shows the experimental setup and behaviors. A neural network controlled the behavior of an autonomous robot and tested in the open-field paradigm. When simulated serotonin levels were high, sensory events led to withdrawn anxious behavior such as wall following and finding its nest (i.e., the robot's charging station). When simulated dopamine levels were high, sensory events led to curious behavior such as locomotion to the middle of the enclosure or exploring a novel object.

**Figure 9:**
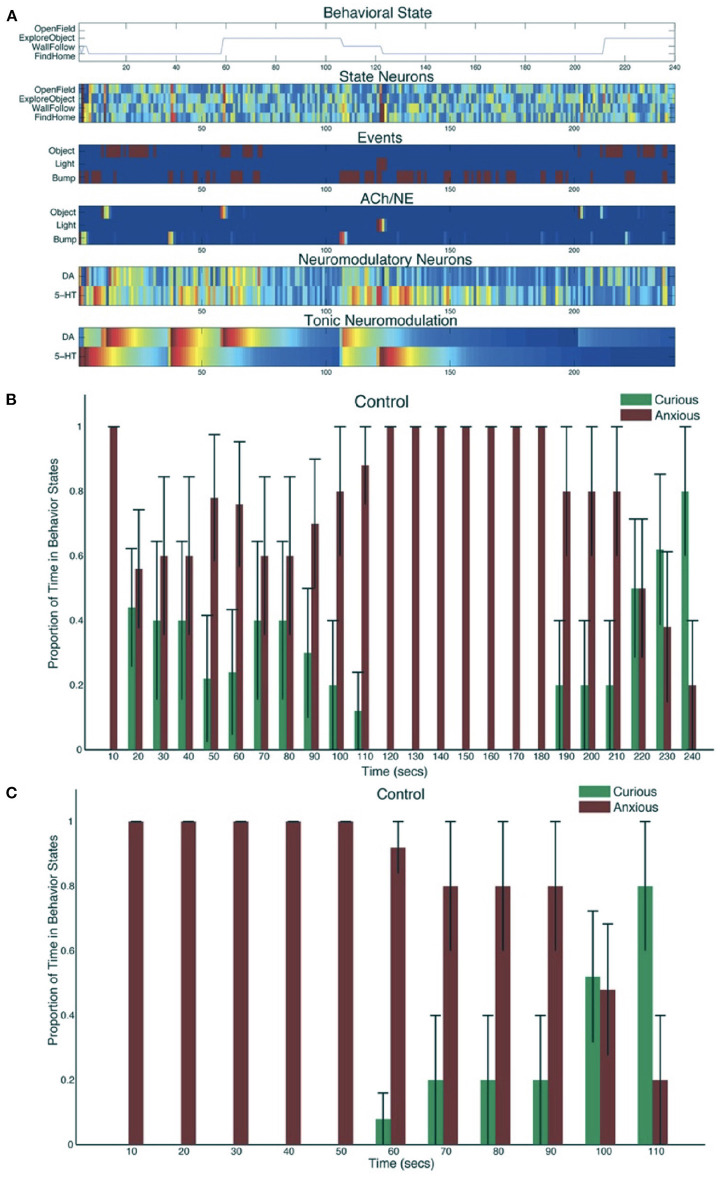
Neurorobot results for invigorated and withdrawn behavior. **(A)** A representative example of behavioral and neural responses in a single trial. The x-axis shows time progression of the trial in seconds. The subplot for “Behavioral State” denotes the state of the robot across time. The subplots for “State Neurons,” “Events,” “ACh/NE,” and “Neuromodulatory Neurons” show neural activity over the trial with pseudocolors ranging from dark blue (no activity) to bright red (maximal activity). The subplot for “Tonic Neuromodulation” denotes the level of tonic activation contributing to DA and 5-HT neurons. **(B)** Proportion of time in Curious (ExploreObject and OpenField) and Anxious (FindHome and WallFollow) behavior, averaged over 5 trials. Error bars represent standard error. **(C)** Similar to B except the behaviors were time-locked to the Light event. Adapted from Krichmar (2013).

The robot responded appropriately to sensory events in its environment. Novel objects resulted in its exploring the environment, and stressful events caused the robot to seek safety. When the environment was unfamiliar, serotonergic activity dominated, resulting in anxious behavior such as WallFollow and FindHome actions. However, once the robot had become more familiar with its environment (approximately 60 s into the trial) DA levels were higher and there was more curious or exploratory behavior. At approximately 120 s into the trial, there was an unexpected light event due to flashing the lights on and off, which resulted in a phasic 5-HT response and a longer tonic increase in 5-HT. This caused the robot to respond with withdrawn or anxious behavior until approximately 210 s into the trial when a pair of object events triggered exploration of the center of the environment. Specifically, tonic levels of 5-HT had decayed, and the object events caused an increase in DA levels triggering a change in behavioral state (see Figure 9A). Figure 9B shows the proportion of curious behavior (OpenField and ExploreObject) and anxious behavior (FindHome and WallFollow) for five experimental trials. Each bar was the average proportion of time spent in either curious (green bars) or anxious (red bars) behaviors. The error bar denoted the standard error. Figure 9C shows the behavior time-locked to the light event. The light event, which occurred at approximately the halfway point in the trial, was introduced to cause a stress response. After the light event, the neurorobot's behavior rapidly switched to anxious behavior until roughly 60 s later when it became curious again. Variation occurred due to different times of the light event and random variations in other sensory events.

Similar to the rodent experiments, changing the serotonin and dopamine levels affected the robot's behavior. Increasing the tonic serotonin levels in the model caused the robot to respond to a stressful event such as a bright light to stay near the walls or its charging station indefinitely. Increasing the tonic DA levels resulted in more curiosity and risk taking. In effect, it took more risks by venturing into the middle of the environment during or right after the stressful light event. Despite the simplicity of the neural network model, the robot's behavior looked quite natural and similar to that of a mouse in the same situation. Since the neuroroboticist was able to precisely control the neuromodulation activity, the experiment could shed light on neurological issues such as anxiety, depression, and obsessive compulsive disorders.

### 4.3. Expected Uncertainty vs. Unexpected Uncertainty

The world is full of uncertainty with which we must cope in our daily lives. Sometimes the uncertainty is expected, forcing us to increase our concentration on a task. Other times the uncertainty is unexpected, forcing us to divert our attention. How we deal with these types of uncertainty can be thought of as a behavioral trade-off. Once again, neuromodulators influence this trade-off of how we apply our attention. Yu and Dayan (2005) suggested that cholinergic neuromodulation tracks expected uncertainty (i.e., the known unreliability in the environment), and noradrenergic neuromodulation tracks unexpected uncertainty (i.e., observations that violate prior expectations) The basal forebrain, where cholinergic neurons reside, encodes the uncertainty of prior information and this can modulate attention to different features. The locus coeruleus, where noradrenergic neurons reside, is involved in cognitive shifts in response to novelty. When there are strong violations of expectations, locus coeruleus activity may induce a “network reset” that causes a reconfiguration of neuronal activity that clears the way to adapt to these changes (Bouret and Sara, 2005). In modeling and in experimental work, it has been shown that the cholinergic system mediates uncertainty seeking (Naude et al., 2016; Belkaid and Krichmar, 2020). Uncertainty seeking is especially advantageous in situations when reward sources are uncertain. The trade-off between expected and unexpected uncertainty can also be observed in how we apply our attention (Avery et al., 2012). Top-down attention or goal-driven attention, which ramps up our attention to look for something, is related to expected uncertainty. Bottom-up or stimulus-driven attention occurs when a surprise or unexpected uncertainty diverts our attention.

**Figure 10:**
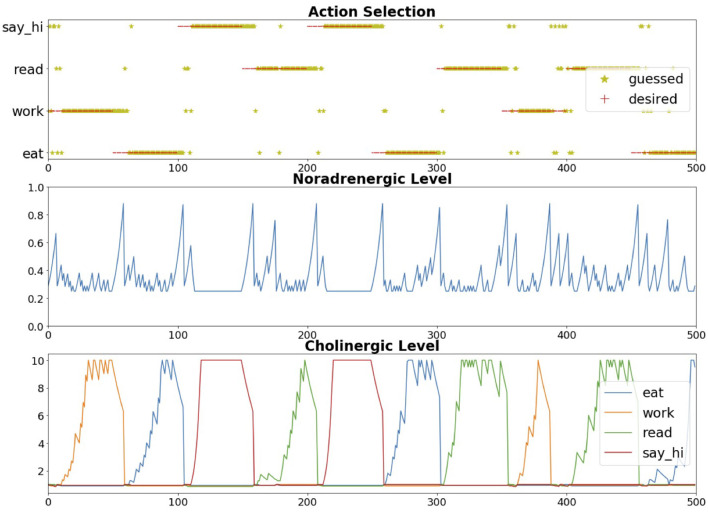
Expected and unexpected uncertainty neuromodulation in neurorobot experiment. Top chart. Robot's response to guess the user's goal. Center chart. Noradrenergic (NE) neuron activity level. Bottom chart. Cholinergic (ACh) neuron activity level. There was an ACh neuron for each potential goal. Adapted from Zou et al. (2020).

The Toyota HSR neurorobot experiment discussed in Section 3.2 and shown in Figure 7 explored this trade-off between expected and unexpected uncertainty by modeling the cholinergic and noradrenergic system ability to regulate attention (Zou et al., 2020). Because the user's goals could be uncertain, simulated cholinergic neurons tracked how likely the user would be to choose any of these goals (i.e., expected uncertainty). When the user interacting with the robot changed their goals (i.e., unexpected uncertainty), the noradrenergic system in the model responded by resetting prior beliefs and rapidly adapting to the new goal. Figure 10 shows how the robot correctly guessed the user's goals, which then drove attention to the object associated with the goal (e.g., eat leads to attention to an apple or orange). Note how quickly the noradrenergic (NE) system responded to goal changes, which led to the cholinergic system (ACh) increasing attention to objects related to the new goal. In this way, the robot was able to monitor a trade-off between the known and unknown uncertainties in the world to rapidly respond to the user's changing needs. Similar to the example in the schema experiments described in Section 3.1, such goal-driven attention could provide benefits for assistive robot technologies.

### 4.4. Exploration vs. Exploitation

During decision making or information gathering, there exists a trade-off between exploration and exploitation in which it is sometimes best to *explore* new options and other times it is best to *exploit* opportunities that have paid off in the past. A framework was presented in which neuromodulation controlled the exploration/exploitation trade-off (Aston-Jones and Cohen, 2005). When neuromodulators have tonic activity, the animal's behavior is exploratory and somewhat arbitrary. However, when the neuromodulator has a burst of phasic activity, the animal is decisive and exploits the best potential outcome at the given time. The CARL robot described in Section 3.2 (see Figure 6) incorporated tonic and phasic neuromodulation (Cox and Krichmar, 2009). When there was tonic neuromodulation, the robot randomly explored its environment by looking at different colored panels. If one of the colors became salient due to a reward or punishment signal, the robot's neuromodulatory systems responded with a phasic burst of activity. This phasic neuromodulation caused a rapid exploitative response to investigate the colored panel. It also triggered learning to approach positive-value objects and avoid negative-value objects.

### 4.5. Foraging vs. Defending

Hormones are chemical messengers in the body that can affect the brain and other organs. They regulate a number of bodily functions such as body temperature, thirst, hunger, and sleep. Like neuromodulators, hormones can be triggered by environmental events and can broadly change neural activity. For example, the hormone orexin regulates hunger levels. This can lead to a behavioral trade-off in which animals foraging for food are less willing to defend a territory (Padilla et al., 2016). Foraging for food may cause an animal to leave its nest exposed to predators. However, defending one's territory requires energy expenditure, which if prolonged requires food for replenishment.

Hormones can be modeled and embodied in robots to explore interesting naturalistic tradeoffs (Canamero, 1997). For example, Cañamero's group modeled hormones that tracked a robot's health (Lones et al., 2018); one hormone was related to the battery level, and another hormone monitored the robot's internal temperature, which was related to how much the robot moved and the climate of its environment. The robot's tasks were to maintain health and gather food resources, which might require aggressive action. This neurorobotics study demonstrated how maintaining health requires behavioral trade-offs. Searching for food increased the robot's internal temperature and reduced the robot's battery level. Being aggressive to obtain food also reduced battery levels. However, not searching for food would lead to starvation. Modeling the secretion and decay of hormones allowed the robot to maintain a comfortable energy and internal temperature level and at times led to an aggressive behavior of pushing objects away to get at food resources. However, as the environments became more complex an epigenetic system, which monitored and controlled the hormones, became necessary for the robot's comfort level to be maintained satisfactorily. Their epigenetic system acted in a similar way to the hypothalamus, a subcortical brain region that regulates many of our bodily functions. Their experiments showed that an epigenetic mechanism significantly and consistently improved the robot's adaptability and might provide a useful general mechanism for adaptation in autonomous robots.

### 4.6. Stress vs. Calm

In his book Why Zebras Don't Get Ulcers, Robert Sapolsky describes how a zebra, which is calmly grazing, responds when it encounters a lion (Sapolsky, 2004). The zebra quickly runs away from this stressful situation. Once clear of danger, the zebra is calm again. This fight-or-flight response is mediated by the stress hormones known as glucocorticoids, which increase blood flow and awareness. However, this stress response does come at the expense of regulating long-term health and short-term memory (Chiba and Krichmar, 2020). Unlike zebras, people sometimes remain in a constant state of stress due to elevated glucocorticoids, which can cause damage to the hippocampus and memory.

Although there has been little work to date on neurorobots that regulate their stress level, downregulation of behavior could be useful for autonomous systems far from power sources, which might have a stress-like response to carry out a mission and then switch to a low-power calm mode after the mission has been accomplished. In an interesting paradigm that explores the stress vs. calm trade-off, experiments have shown that rats appear to be capable of empathy and prosocial behavior (Ben-Ami Bartal et al., 2011). In one study a rat was trapped in a cage and clearly stressed. Another rat observing this behavior became stressed, too. The observing rat, feeling bad for its trapped friend, found a lever that opened the cage and released the trapped rat. This study suggested that rats can feel another's pain (i.e., feel empathy) and are willing to act on the other's behalf (i.e., can be prosocial).

In a robotic variation of the empathy experiment, a rat was trapped in a cage interacted with two different robots, one of which was helpful and opened the cage and the other of which was uncooperative and ignored the trapped rat (Quinn et al., 2018). Interestingly, the rat remembered who its robot friends were. When the helpful robot was trapped, the rat freed that robot but did not free the robot that was uncooperative. This could have implications for rescue robotics. A robot that can identify and relieve stress or anxiety could have applications for robotic caretakers or for disaster relief.

### 4.7. Social vs. Solitary

Hormones can regulate a trade-off between social bonding and independence. Estrogen, progesterone, oxytocin, and prolactin can influence a number of neural systems to ensure maternal nurturing, bonding, and protection of young (Rilling and Young, 2014). In particular, oxytocin has been shown to regulate social and paternal bonding (Young and Wang, 2004). An interesting example of oxytocin's effect on bonding has been observed in voles. Whereas, prairie voles are polygamous and the male does not assist in the nurturing of young pups, meadow voles are monogamous and both parents participate in the pup rearing. Interestingly, meadow voles have more oxytocin receptors than prairie voles. Furthermore, inhibiting oxytocin prevents pair bonding in meadow voles (Young and Wang, 2004). However, social bonding requires devoting energy to another, possibly at the expense of one's own health. Therefore, it could be argued that there should be a balance between social and solitary behavior.

Neurorobot experimenters have investigated the balance between social and solitary behavior. By simulating social hormones, Cañamero's group investigated attachment bonds between a robot and a “caregiver” (Canamero et al., 2006; Hiolle et al., 2009, 2012). Although they did not explicitly model oxytocin, their system did simulate the type of bonding observed between a parent and a child. The robot found a good balance between asking the caregiver for help and learning on its own. Too much interaction with a caregiver led to stress and rejection by the robot. Not enough interaction with a caregiver resulted in isolation. As with humans, a proper balance is important for learning and development.

## 5. Discussion

In this article, we discussed a number of principles to consider when designing neurorobots and experiments using robots to test brain theories. These principles are strongly inspired by Pfeifer and Bongard's design principles for intelligent agents. We build upon these design principles by grounding them in neurobiology and by adding principles based on neuroscience research. We highlight the importance in neurorobotics for designing systems that are reactive, adaptive, predictive, able to manage behavioral tradeoffs, and capable of learning from experience.

**Figure 11:**
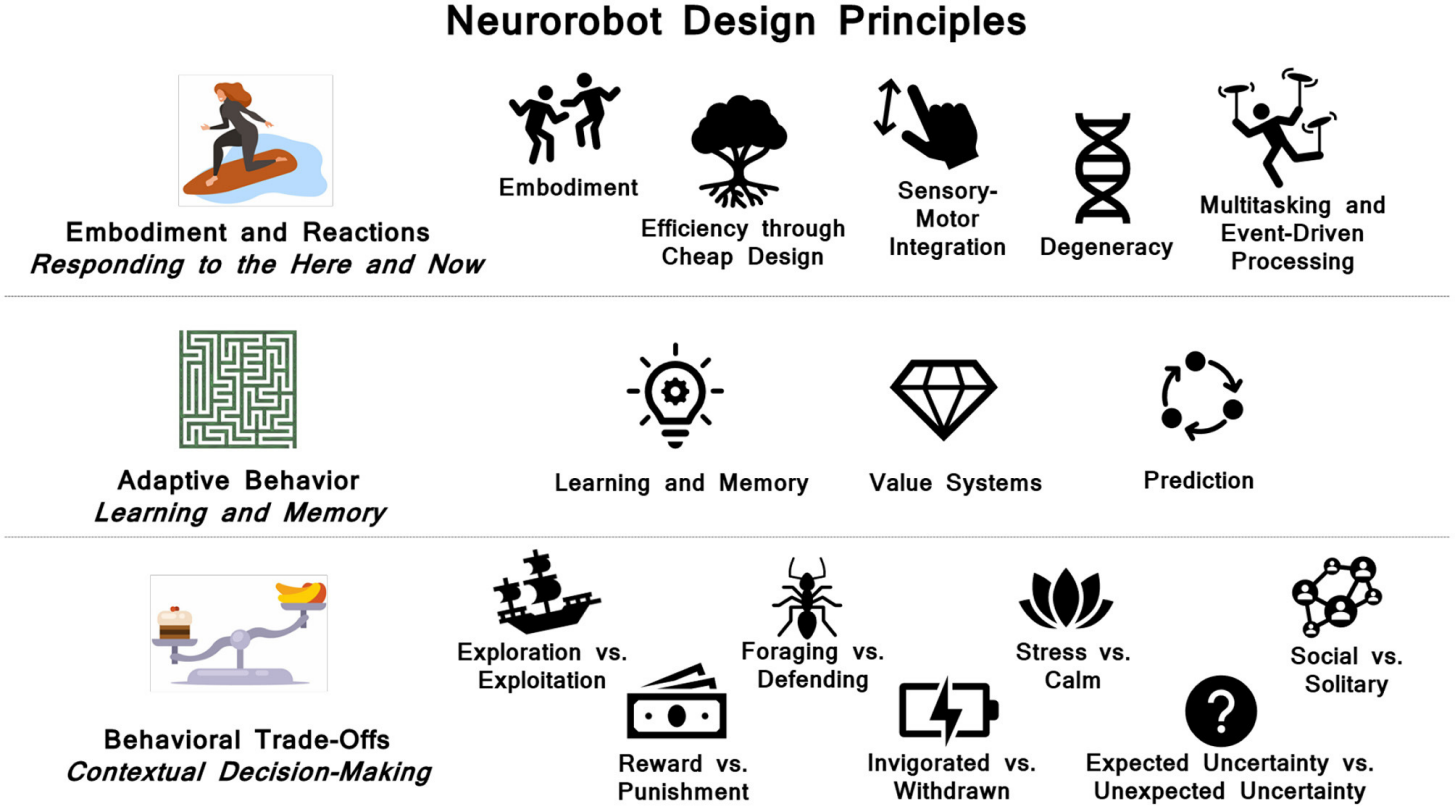
Summary of neurorobot design principles.

As summarized in Figure 11, the principles fall into three broad categories: 1) *Embodiment and reactions*. These are reactive, reflexive and rapid responses. They are often carried out without involving the central nervous system. Rather they emerge through the body's interaction with the environment or are handled by the peripheral nervous system and reflex arcs involving the spinal cord. They can have short-term adaptive properties and lead to behavioral repertoires. 2) *Adaptive behavior*. Biological organisms have the ability to learn continually over the lifetime of the organism. A major property of the brain is its plasticity. In particular, hippocampal interactions with the cortex lead to long-term contextual memory. Neuromodulatory systems can signal value, which shapes learning and triggers adaptive behavior. Another hallmark of the brain is its ability to predict outcomes. This requires the construction, maintenance and updating of memory systems. 3) *Behavioral trade-offs*. To survive in a dynamic world, organisms must make decisions based on their needs and environmental context. Oftentimes, these needs are a trade-off between opposing motivations (e.g, taking a risk for a reward vs. playing it safe to avoid punishment). These trade-offs can lead to interesting behavior. Many of these trade-offs are regulated by sub-cortical neuromodulator and hormone levels.

### 5.1. Importance of Low-Level Processes and Model Organisms

Although the examples in this article focused primarily on vertebrates, the principles could be applied to modeling other organisms. Studies of the insect visual system have led to elegant, efficient solutions for robot navigation that could be deployed on neuromorphic hardware (Galluppi et al., 2014; Schoepe et al., 2021). The emphasis on vertebrates and especially the mammalian brain has been data-driven in part. There are numerous studies of rodents, non-human primates and humans that have provided modelers with anatomical, behavioral, and neurophysiological data to make their simulations more biologically accurate. However, the complexity of these organisms and their brains makes holistic modeling difficult. A promising avenue may be to study organisms that have less complex brains and behaviors. Recent work on model organisms such as drosophila and the nematode C. Elegans are providing rich data sets for neuroboticists (Sarma et al., 2018; Scheffer et al., 2020). The OpenWorm project (Sarma et al., 2018) provides the biological data and the simulation tools, including a robotics sub-project, to create interesting neurorobots that follow many of the design principles introduced here.

There is a tendency in neurorobotics, which is a subarea of cognitive robotics (Cangelosi and Asada, 2022), to model cognitive functions such as attention, decision-making, planning, etc. Although modeling human cognition may be an ultimate goal for the field, many of the design principles introduced here concentrate on low-level processes such as how the body shapes behavior, motivations, homeostatic control of body functions to name a few. As we emphasized in this paper and in Chiba and Krichmar (2020), many of these processes are driven by sub-cortical brain regions and neurochemicals. Their interaction with the environment and bodily functions lead to interesting behavior that could be described as cognitive. Early examples of neurorobots and behavior-based robots demonstrated that intelligent behavior could arise from interactions between the robot and the environment without complex nervous systems or control systems (Braitenberg, 1986; Brooks, 1991; Holland, 2003). More work needs to be done to first build a foundation of these low-level processes, upon which higher-order cognitive processes can be added. Moreover, each cognitive model should carefully choose an appropriate level of abstraction and state assumptions about the lower level processes that support the model.

### 5.2. Next Steps and Future Directions

The principles of neurorobotics introduced here can address major challenges facing artificial intelligence and robotics research. In general, neurorobotics explores these challenges in embodied settings, providing a fresh perspective that extends beyond simulations and algorithms performed on computers. Neurorobotics incorporates features of the brain that may begin to address lifelong continual learning, efficient computing, operating on scarce knowledge, and human-computer interaction.

We expect that new neuroscience discoveries will further inform neurorobots, and vice versa. Progress in learning and memory may lead to applications capable of continual learning. Advances in our understanding of multimodal sensory systems may be incorporated into neurorobotics that not only classify but also understand the meaning of what they are perceiving. Given the recent achievements of artificial intelligence, hybrid systems combining machine learning and deep learning with neurorobotic design principles could lead to interesting applications in autonomous driving, assistive robots, and manufacturing.

In the intermediate term, we believe that neurobiological concepts in learning and memory, navigation, decision making, social behavior, and more, will have found their way into practical applications. Progress in neuromorphic computing and algorithms will lead to applications that can run at the edge with little human intervention. This may lead to advances in search and rescue robots and in robots capable of autonomously exploring unknown environments such as the deep sea or extraterrestrial planets.

In the long-term we hope that neurorobotics will achieve more general intelligence rather than being designed for specific tasks. In fact the delineation between conventional robotics and neurorobotics may be blurred, with all robots possessing some neurobiologically inspired aspects. With the rapid advances in computing and other technologies, it is hard to predict far into the future. However, we do believe that neurorobotics and cognitive machines, in some form, will seamlessly be a part of our everyday lives.

## 6. Conclusion

In closing, we believe that neurorobots that follow many of the design principles discussed in this article will have more interesting, naturalistic behavior. This not only allows the robot to be a better model for understanding the complex behaviors observed in biology, it also could lead to better robots that show more intelligence and that are more natural in their interactions with other agents.

## Data Availability Statement

The raw data supporting the conclusions of this article will be made available by the authors, without undue reservation.

## Author Contributions

All authors listed have made a substantial, direct, and intellectual contribution to the work and approved it for publication.

## Funding

This work was supported by NSF award IIS-1813785 and by the United States Air Force Award FA9550-19-1-0306.

## Conflict of Interest

TH was employed by Riot Games. The remaining author declares that the research was conducted in the absence of any commercial or financial relationships that could be construed as a potential conflict of interest.

## Publisher's Note

All claims expressed in this article are solely those of the authors and do not necessarily represent those of their affiliated organizations, or those of the publisher, the editors and the reviewers. Any product that may be evaluated in this article, or claim that may be made by its manufacturer, is not guaranteed or endorsed by the publisher.
